# Directional Phase and Polarization Manipulation Using Janus Metasurfaces

**DOI:** 10.1002/advs.202406571

**Published:** 2024-08-09

**Authors:** Yiwen Zhou, Teng Zhang, Guannan Wang, Ziqing Guo, Xiaofei Zang, Yiming Zhu, Fei Ding, Songlin Zhuang

**Affiliations:** ^1^ Terahertz Technology Innovation Research Institute and Shanghai Key Lab of Modern Optical System University of Shanghai for Science and Technology No. 516 JunGong Road Shanghai 200093 China; ^2^ Centre for Nano Optics University of Southern Denmark Campusvej 55 Odense M DK‐5230 Denmark

**Keywords:** directional imaging, directional phase and polarization modulation, direction‐ and polarization‐dependent detection, Janus metasurfaces

## Abstract

Janus metasurfaces, exemplifying two‐faced 2D metamaterials, have shown unprecedented capabilities in asymmetrically manipulating the wavefront of electromagnetic waves in both forward and backward propagating directions, enabling novel applications in asymmetric information processing, security, and signal multiplexing. However, current Janus metasurfaces only allow for directional phase manipulation, hindering their broader application potential. Here, the study proposes a versatile Janus metasurface platform that can directionally control the phase and polarization of terahertz waves by integrating functionalities of half‐wave plates, quarter‐wave plates, and metallic gratings within a cascaded metasurface structure. As a proof‐of‐principle, the study experimentally demonstrates Janus metasurfaces capable of independent and simultaneous control over phase and polarization, showcasing propagation direction‐encoded focusing and polarization conversion. Moreover, the directionally focused points are utilized with distinct polarization states for advanced applications in direction‐ and polarization‐sensitive detection and imaging. This unique strategy for simultaneous phase and polarization control with direction‐dependent versatility opens new avenues for designing ultra‐compact devices with significant implications in imaging, encryption, and data storage.

## Graphical abstract

A versatile Janus metasurface platform is proposed and experimentally demonstrated to directionally control the phase and polarization of THz waves, leading to the generation of one and two focal points with different polarization states in the forward and backward directions. Furthermore, the direction‐ and polarization‐dependent detection and imaging are realized based on the designed Janus metasurfaces.

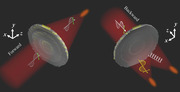

## 1 Introduction

Metasurfaces, 2D counterparts of metamaterials, have emerged as an ultracompact and flexible platform for manipulating the wavefront of electromagnetic (EM) waves, leading to the development of miniaturized optical components with unprecedented functionalities.^[^ ^1^, ^2^ ^]^ Benefiting from the exotic properties of metasurfaces, numerous applications have been demonstrated, including beam steering,^[^ ^3^, ^4^, ^5^, ^6^, ^7^, ^8^ ^]^ polarization converters,^[^ ^9^, ^10^, ^11^, ^12^, ^13^, ^14^, ^15^ ^]^ vortex generators,^[^ ^16^, ^17^, ^18^, ^19^, ^20^, ^21^ ^]^ and holograms.^[^ ^22^, ^23^, ^24^, ^25^, ^26^, ^27^ ^]^ Among a plethora of advantages of metasurfaces, such as ultrathin nature, ease of design and fabrication, local wavefront manipulation, and multifunctional integration, the capability of multiplexing multiple functions within a single metasurface is particularly desirable for compact photonic systems. In particular, polarization‐multiplexing,^[^ ^28^, ^29^, ^30^, ^31^, ^32^, ^33^, ^34^, ^35^, ^36^ ^]^ wavelength‐multiplexing,^[^ ^37^, ^38^, ^39^ ^]^ and incident angle‐multiplexing^[^ ^40^, ^41^ ^]^ have been implemented. Nevertheless, exploring new degrees of freedom (DoFs) for further integrating multiple functions into a single metasurface remains crucial for advancing integrated systems.

Apart from polarization, wavelength, and incident angle, the propagation direction has emerged as a new DoF to implement Janus metasurfaces with distinct functions on each side,^[^ ^42^, ^43^, ^44^, ^45^ ^]^ different from conventional metasurfaces that exhibit identical properties regardless of the direction of incident waves. For instance, a directional Janus metasurface composed of cascaded anisotropic impedance sheets was designed to realize asymmetric transmission with different phase distributions in the forward and backward directions.^[^ ^42^ ^]^ Additionally, dual‐layer metasurfaces combining geometric metasurfaces and metallic gratings have been used to generate asymmetric holograms^[^ ^43^ ^]^ and one‐way focusing,^[^ ^44^ ^]^ respectively. Furthermore, integrating phase change materials has led to the development of thermally activated Janus metasurfaces with switchable, directional phase functions.^[^ ^45^ ^]^ In addition, the Janus reflective polarization‐division metadevices for beam steering, directionally scattering response, i.e., super radome with asymmetric diffusion and absorption, and spin‐encoded wavelength‐direction multitasking Janus metasurfaces have been proposed and experimentally demonstrated.^[^ ^46^, ^47^, ^48^ ^]^ Despite these achievements, existing Janus metasurfaces are primarily limited to phase modulation in opposite propagation directions, inevitably hindering practical applications. Polarization, like phase, is a fundamental property of EM waves and can be used for processing, recording, and storing information. However, realizing directional polarization and phase modulation simultaneously remains a significant challenge. Moreover, previous works mainly focus on the asymmetric manipulation of the phase of EM waves without considering the practical applications. To further explore the applications of asymmetric metasurfaces is of great importance.

In this paper, we propose a novel approach to design cascaded Janus metasurfaces that break spatial symmetry in opposite directions, resulting in distinct phase and polarization functions. Our Janus metasurface, comprising anisotropic meta‐atoms functioning as half‐wave and quarter‐wave plates (HWPs and QWPs) along with metallic gratings with polarization selective response, enables the directional phase and polarization manipulation, generating one and two focal points with different polarization states in the forward and backward directions. To reveal the practical applications of our designed metasurfaces, we further experimentally demonstrate direction‐ and polarization‐dependent detection and imaging using our designed Janus metasurfaces. We further experimentally demonstrate direction‐ and polarization‐dependent detection and imaging using our designed Janus metasurfaces. The unique and robust approach for controlling both phase and polarization in the propagation direction domain opens new possibilities for high‐capacity data storage and information encryption.

## 2 Results

### 2.1 Design of Janus Metasurfaces for Directionally Controlling Phase and Polarization

**Figure 1:**
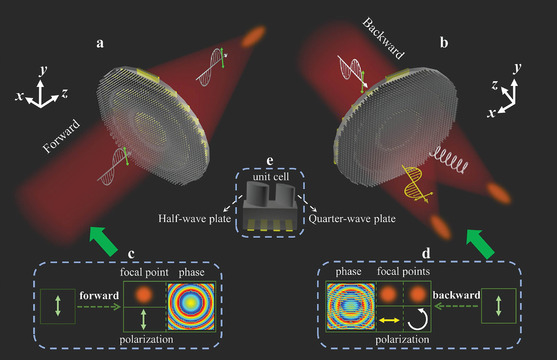
Schematics of a cascaded Janus metalens for directional and simultaneous manipulation of phase and polarization. a) Under forward incidence, a focal point is generated with the same polarization as the incident THz waves. b) Under backward incidence, two focal points are created with orthogonal and circular polarizations relative to the incident THz waves. c) Schematic of an *y*‐polarized focal point with the focusing phase profile when illuminated by *y*‐polarized THz waves from the forward direction. d) Schematic of *x*‐ and circularly‐polarized focal points with the focusing phase profile when illuminated by *y*‐polarized THz waves from the backward direction. e) Unit cell consisting of a HWP, a QWP, and metallic gratings.

**Figure** **1** schematically shows a Janus metasurface that directionally manipulates the phase and polarization of linearly‐polarized (LP) THz waves illuminated from both forward and backward directions. This Janus metasurface comprises a variety of complex meta‐atoms, each consisting of a half‐wave plate (HWP), a quarter‐wave plate (QWP), and metallic gratings. The wave plates (two ellipse microrods in a unit cell) that transform polarization states differ in size but share the same rotation angle. The metallic gratings function as a linear polarizer that transmits *y*‐polarized waves and reflects *x*‐polarized waves. Upon illumination with *y*‐polarized THz waves from the forward direction, a focal point with the same polarization is observed after the cascaded Janus metasurface (Figure 1a). In contrast, for the backward incidence of *y*‐polarized THz waves, two focal points are generated: one *x*‐polarized focal point and one left‐handed circularly polarized (LCP) focal point (Figure 1b). Therefore, the phase distributions and polarization states are completely different in the forward and backward directions (Figure 1c,d), allowing the proposed Janus metasurface to achieve directional and simultaneous manipulation of phase and polarization in opposite directions. Figure 1c,d show phase profiles, electric‐field intensity distributions, and polarization states of the generated focal points when the *y*‐polarized THz wave illuminates from forward and backward directions. The forward and backward focusing are obtained by rotating the designed metasurfaces while fixing the direction of the incident THz waves.

To realize directional phase and polarization manipulation, HWP and QWP meta‐atoms (Figure 1e) should possess both focusing and polarization conversion functionalities. For a focal point located at (*x_i_*, *y_i_*, *f_i_*), the metasurface should incorporate the hyperbolic phase profile $\varphi \left(x,y\right)\;=\frac{2\pi }{\lambda }\;\sqrt{{\left(x-x_{i}\right)}^{2}+{\left(y-y_{i}\right)}^{2}+{f_{i}}^{2}}-f_{i}$, where *f_i_* is the focal length, (*x_i_*, *y_i_*,) is the local coordinate on the metasurface plane, and *λ* is the working wavelength. The phase profile is discretized and represented by designed HWP and QWP meta‐atoms. Besides focusing, HWP meta‐atoms allow for the rotation of linear polarization (Section S1, Supporting Information). When *x*‐polarized THz waves interact with HWP meta‐atoms with the uniform in‐plane orientation of *θ* = 45° with respect to the *x*‐axis, the polarization‐rotated angle of the converted THz waves is 2*θ*, corresponding to *y*‐polarized waves. When the QWP meta‐atoms are uniformly rotated with an angle of *θ* = 45°, transformations between LP and circularly polarized (CP) THz waves are enabled (Section S1, Supporting Information).

**Figure 2:**
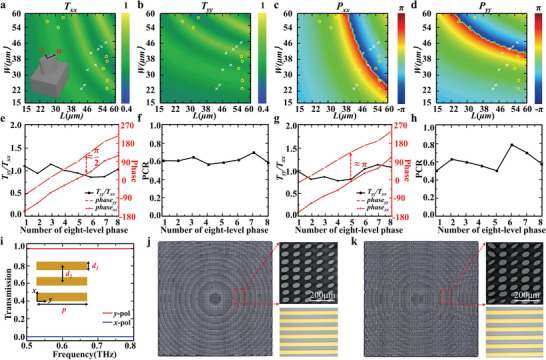
Design and fabrication of meta‐atoms. a–d) Simulated (a,b) transmittance and (c,d) phase maps as a function of lateral dimensions for (a,c) *x*‐ and (b,d) *y*‐polarized incidence. e) The transmittance ratios (black curve) and phases (red curves) of the selected QWP meta‐atoms between *x*‐ and *y*‐polarized incidence. f) Polarization conversion ratios between transmitted CP THz waves and the incident LP THz waves of the selected QWP meta‐atoms. g) The transmittance ratios (black curve) and phases (red curves) of the selected HWP meta‐atoms between *x*‐ and *y*‐polarized incidence. h) Polarization conversion ratios between the transmitted cross‐polarized CP THz waves and the incident CP THz waves of the selected HWP meta‐atoms. i) Transmission spectra of metallic gratings (inset) under the illumination of *x*‐ and *y*‐polarized THz waves, respectively. j) Optical images of a cascaded Janus metasurface consisting of QWP meta‐atoms and metallic gratings. k) Optical image of a cascaded Janus metasurface consisting of QWP and HWP meta‐atoms as well as metallic gratings. Insets in the top right corner of (j,k) are zoom‐in images of meta‐atoms, while insets in the bottom right corner of (j,k) are zoom‐in images of metallic gratings.

To design HWP and QWP functionalities, we deliberately studied the transmission properties of elliptic meta‐atoms with different dimensions. The lattice constant of a meta‐atom is 130 µm, which is small enough to avoid any diffraction at the designed working frequency of 0.72 THz. The long axis of each meta‐atom is along the diagonal or anti‐diagonal direction. The thicknesses of silicon substrate and microrods are 600 and 400 µm, respectively. After optimization, we selected sixteen ellipse microrods to realize the functionalities of HWPs (circular rings in **Figure** **2**a–d) and QWPs (crosses in Figure 2a–d). The structural parameters of selected meta‐atoms are given in Tables S1 and S2 (Supporting information). As shown in Figure 2e,g, the transmittance ratios between *x*‐ and *y*‐polarized THz waves approach 1, while the relative phase differences are about π/2 and π, respectively, demonstrating QWP and HWP elements with good performance. Moreover, the efficiency of polarization conversion of QWPs (HWPs) ranges from 58% (52%) to 70% (74%), as shown in Figure 2f,h. The reduced efficiency primarily arises from reflections off the silicon substrate and the design criteria of multiple QWPs and HWPs. By integrating metallic gratings (Table S3, Supporting Information) on the other side of the silicon substrate, directional phase and polarization manipulation can be realized due to their polarization‐selective transmission property: under the illumination of *y*‐polarized (*x*‐polarized) THz waves, the metallic gratings can completely transmit (reflect) the incident beam (Figure 2i).

### 2.2 Single‐Focal Janus Metalens for Directional Focusing and Polarization Conversion

**Figure 3:**
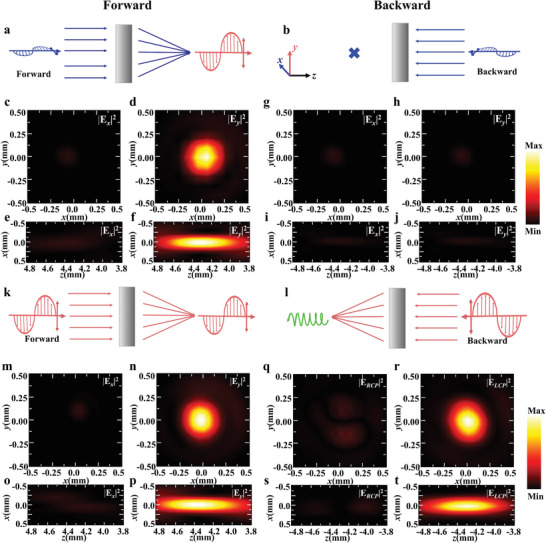
Single‐focal Janus metalens for directional focusing and polarization conversion. a,b) Schematics of one‐way focusing under the incidence of *x*‐polarized THz waves. c–f) The measured electric‐field intensity distributions (|*E_x_* |^2^ and | *E_y_* |^2^) at the focal plane (c,d) or the *x*‐*z* plane (e,f) for forward incidence of *x*‐polarized THz waves. g–j) The measured electric‐field intensity distributions (|*E_x_* |^2^ and | *E_y_* |^2^) at the focal plane (g,h) or the *x*‐*z* plane (i,j) for backward incidence of *x*‐polarized THz waves. k,l) Schematics of focusing and directional polarization manipulation for the forward (k) and backward (l) incidence of *y*‐polarized THz waves. m–p) The measured electric‐field intensity distributions (|*E_x_* |^2^ and | *E_y_* |^2^) at the focal plane (m,n) or the *x*‐*z* plane (o,p) for forward incidence of *y*‐polarized THz waves. q–t) The measured electric‐field intensity distributions (|*E_x_* |^2^ and | *E_y_* |^2^) at the focal plane (q,r) or the *x*‐*z* plane (s,t) for backward incidence of *y*‐polarized THz waves.

To verify our proposed approach, we first designed and fabricated a cascaded single‐focal metalens (Figure 2j) for directional focusing and polarization conversion by switching the polarization of the incident THz waves, as shown in **Figure** **3**. This single‐focal Janus metalens consists of 100 × 100 QWP meta‐atoms and polarization‐selective metallic gratings. The focal length is predesigned as 4.4 mm at the frequency of 0.72 THz. Figure 3a,b schematically show one‐way focusing under forward‐propagating *x*‐polarized THz waves, while backward‐propagation *x*‐polarized waves are blocked. The measured electric‐field intensity distributions indicate that only one *y*‐polarized focal point is observed at *z* = 4.3 mm away from the metallic gratings for forward incidence of *x*‐polarized THz waves (Figure 3c,d), which are in good agreement with simulation results in Section S3 (Supporting information). For the forward incidence, the *x*‐polarized THz waves interact with QWP meta‐atoms and are converted to a CP focusing beam, which can be decomposed into *x*‐ and *y*‐polarized components with identical amplitudes. The *y*‐polarized component is transmitted through metallic gratings due to the polarization selection function, resulting in a *y*‐polarized focal point with a measured efficiency of ≈16.1% (Table S4, Supporting information). The measured intensity distributions in the *x*‐*z* plane are shown in Figure 3e,f. In contrast, no focal point is observed after the meta‐atoms for the backward‐propagating *x*‐polarized THz waves (Figure 3g–j), since the backward THz waves are completely reflected by metallic gratings. Thus, this cascaded single‐focal metalens shows only directional phase modulation for *x*‐polarized incident THz waves from forward and backward directions. When this metalens is illuminated by *y*‐polarized THz waves, the directional polarization conversion can be excited from opposite directions, as shown in Figure 3k,l. For the forward incidence of *y*‐polarized THz waves, a *y*‐polarized focal point located at (0, 0, 4.3 mm) is experimentally measured, as shown in Figure 3m–p. In contrast, when *y*‐polarized THz waves are illuminated from the backward direction, a focal point is still generated away from the meta‐atoms (Figure 3q–t). This focal point is located at (0, 0, −4.3 mm), which is symmetric to the focal point generated for the forward incidence, demonstrating identical focusing phases for these two focal points. However, the polarizations of these two focal points are different, with a *y*‐polarized focal point and an LCP focal point for the *y*‐polarized incidence from opposite directions, resulting in higher focusing efficiency (Table S4, Supporting Information). Therefore, directional polarization conversion can be realized in the designed metalens by switching the *x*‐polarized incidence into the *y*‐polarized incidence. The directional characteristics of this single‐focal metalens under CP incidence are supplied in Section S5 (Supporting Information). To verify the versatility of our concept, we designed a similar cascaded metasurface consisting of HWPs and metallic gratings and discussed its performance in Section S6 (Supporting Information).

### 2.3 Dual‐Focal Janus Metalens for Directional Focusing and Polarization Conversion

**Figure 4:**
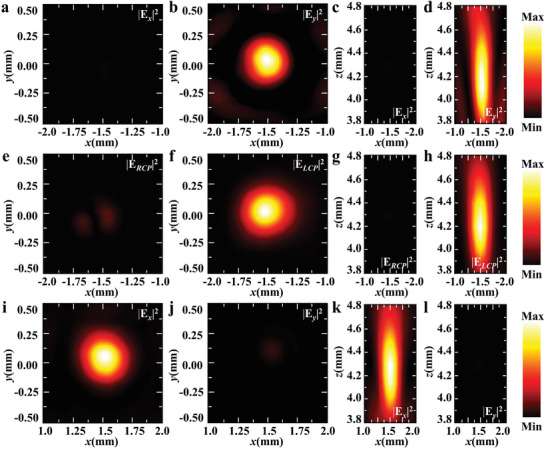
Dual‐focal Janus metalens for directional focusing and polarization conversion. a–d) The measured electric‐field intensity distributions (|*E_x_* |^2^ and | *E_y_* |^2^) at the focal plane (a,b) or the *x*‐*z* plane (c,d) for the forward incidence of *y*‐polarized THz waves. e–h) The measured electric‐field intensity distributions (|*E_LCP_* |^2^ and | *E_RCP_* |^2^) at the focal plane (e,f) or the *x*‐*z* plane (g,h) for the backward incidence of *y*‐polarized THz waves. i–l) The measured electric‐field intensity distributions (|*E_x_* |^2^ and | *E_y_* |^2^) at the focal plane (i,j) or the *x*‐*z* plane (k,l) for the backward incidence of *y*‐polarized THz waves.

To further show the versatility of our platform, we fabricated a dual‐focal Janus metalens that encodes the functionalities of an HWP, a QWP, and metallic gratings to realize directional focusing and asymmetric polarization conversion (Figure 2k). **Figure** **4**a,b show measured electric‐field intensity distributions (|*E_x_* |^2^ for Figure 4a and |*E_y_* |^2^ for Figure 4b) for the forward incidence of *y*‐polarized THz waves. A *y*‐polarized focal point located at (−1.5 mm, 0, 4.3 mm) is generated away from the metallic gratings. The measured electric‐field intensity distributions in the *x*‐*z* plane are also shown in Figure 4c,d, respectively. For *y*‐polarized incident THz waves in the forward direction, they first interact with the HWP and QWP meta‐atoms. Consequently, one part of the incident THz wave becomes an *x*‐polarized focusing beam, while another part is converted into a CP focusing beam. Since the metallic gratings filter out all *x*‐polarized components in transmission, only the *y*‐polarized component of the CP focusing beam passes through the metallic gratings, resulting in a *y*‐polarized focal point with an efficiency of ≈10.4% (Table S5 and Section S7, Supporting Information) for the forward incidence. In contrast, when *y*‐polarized THz waves are illuminated from the backward direction, two focal points with different polarization states are generated, as shown in Figure 4e–l. For the backward incidence of *y*‐polarized THz waves, the metallic gratings are transparent, allowing the transmitted THz waves to be converted into an LCP focal point and an *x*‐polarized focal point due to the QWP and HWP meta‐atoms, respectively. As such, a single *y*‐polarized focal point is observed for the forward incidence, while double focal points with circular and linear polarization states are generated for the backward incidence, with a higher efficiency of ≈20.8% (Table S5 and Section S7, Supporting Information). This results in different phase functions and polarizations in the forward and backward directions, demonstrating directional manipulation of phase and polarization in opposite directions. The numerical simulations of the dual‐focal metalens are given in Section S8 (Supporting information), which match well with experimental measurements. The directional characteristics of this dual‐focal metalens under CP and *x*‐polarized incidence are shown in Section S9 (Supporting information). Besides regular Gaussian beams, focused vortex beams with direction‐controlled polarization states can be accordingly designed (Sections S10 and S11, Supporting information).

### 2.4 Direction‐ and Polarization‐Dependent Detection and Directional Imaging

**Figure 5:**
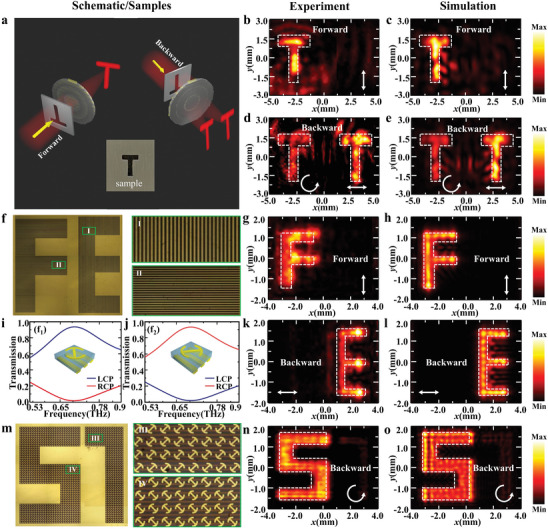
Dual‐focal Janus metalens for directional imaging as well as direction‐ and polarization‐dependent detection. a) Schematic of directional imaging. Inset is the imaging sample consisting of a letter “T”. b,c) The measured and simulated imaging for the incidence of THz waves from the forward direction. d,e) The measured and simulated imaging for the incidence of THz waves from the backward direction. f) LP‐dependent target. g,h) The measured (g) and simulated (h) imaging of the LP‐dependent target for the forward incidence. i,j) Transmission spectra of two dual‐layer chiral‐structures. k,l) The measured (k) and simulated (l) imaging of the LP‐dependent target for the backward incidence. m) CP‐dependent target. n,o) The measured (n) and simulated (o) imaging of the CP‐dependent target for the backward incidence.

The proposed Janus metasurface not only realizes different phase functions in opposite directions but also enables directional control of polarization states. Capitalizing on the dual‐focal Janus metalens, we demonstrate direction‐ and polarization‐dependent detection as well as directional imaging in the THz region (**Figure** **5**), which holds potential for applications in pattern recognition and bio‐imaging. Figure 5a shows the schematic of directional imaging, in which a single letter or dual letters are imaged in the forward or backward directions. The object to be imaged is an air slit of letter “T” milled on a stainless‐steel sheet (inset in Figure 5a). For the incidence of *x*‐polarized THz waves from the forward direction, one single image of the “T” is reconstructed after the metasurface, as shown in Figure 5b. On the contrary, two images of the “T” are formed when *x*‐polarized THz waves are illuminated from the backward direction (Figure 5d). The calculated results in Figure 5c,e are reasonably matched with the measurements. In addition to directional imaging, the designed dual‐focal Janus metalens also enables direction‐ and polarization‐dependent detection. As shown in Figure 5f, two capital letters “F” and “E”, consisting of metallic gratings with long axis along the *x*‐ and *y*‐axis, are designed and fabricated for direction‐ and LP‐dependent detection. For the forward incidence of *y*‐polarized THz waves, a *y*‐polarized focal point is generated after the metallic gratings. When this LP‐dependent target is placed in the region of this *y*‐polarized focal point, the capital letter “F” is revealed by scanning the target while the THz detector and metasurface remain fixed, as shown in Figure 5g. The experimental result agrees well with the numerical simulation, as shown in Figure 5h. When the LP‐dependent target is positioned at (1.5 mm, 0, −4.3 mm) and the metasurface is illuminated from the backward direction with *y*‐polarized THz waves, the capital letter “E” is revealed by scanning the target (Figure 5k), which is matched with the simulated result (Figure 5l). For the case of backward incidence, another LCP focal point is generated. To demonstrate CP‐dependent detection, a cascaded target consisting of meta‐atoms with split ring meta‐atoms and metallic gratings is designed (right figures in Figure 5m), where the helicity‐dependent characteristics are numerically shown in Figure 5i,j. When the opening orientation of the meta‐atoms is along the anti‐diagonal direction (Figure 5i), LCP THz waves near 0.72 THz can be transmitted through the designed target (i.e., “5” in Figure 5m), while RCP THz waves near 0.72 THz can be transmitted through the designed target (i.e., “7” in Figure 5m) for the opening orientation along the diagonal direction (Figure 5j). When the CP‐dependent target that consists of two numbers “5” and “7” is located at (−1.5 mm, 0, −4.3 mm), only the number “5” is visible in the experiment (Figure 5n), which is in good agreement with the simulated result (Figure 5o), indicating the helicity‐dependent detection. The detailed discussions of imaging efficiency are shown in Section S12 (Supporting Information).

## 3 Discussion and Conclusion

**Figure 6:**
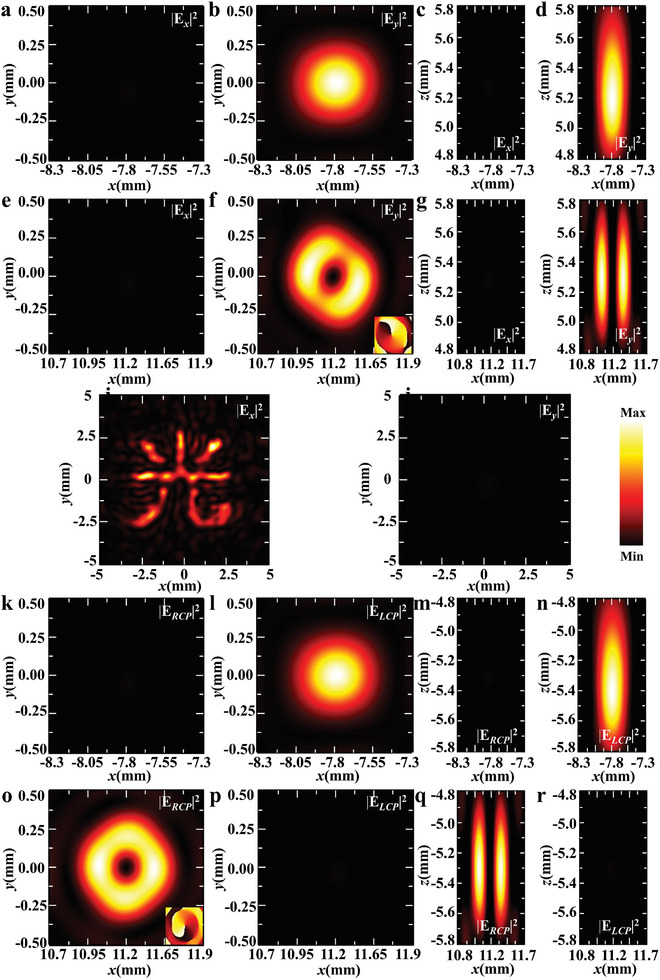
Janus metasurface for simultaneously controlling phase and polarization for directional focusing and polarization conversion. a–d) and e–h) The simulated electric‐field intensity distributions (|*E_x_* |^2^ and | *E_y_* |^2^) at the focal plane (a,b,e,f) or the *x*‐*z* plane (c,d,g,h) for the forward incidence of the *y*‐polarized THz waves. i,j) The simulated electric‐field intensity distributions (|*E_x_* |^2^ and | *E_y_* |^2^) for the backward incidence of the *y*‐polarized THz waves. k–n) and o–r) The simulated electric‐field intensity distributions (|*E_LCP_* |^2^ and | *E_RCP_* |^2^) at the focal plane (k,l,o,p) or the *x*‐*z* plane (m,n,q,r) for the backward incidence of the *y*‐polarized THz waves.

To further extend the versatile functionalities of our proposed approach, we numerically demonstrate additional channels for directionally manipulating the phase and polarization of THz waves in **Figure** **6**. Unlike the previously used QWP meta‐atoms that can only transform *x*‐polarized THz waves into LCP THz waves, we introduce additional QWP meta‐atoms capable of transforming *x*‐polarized THz waves into RCP THz waves. The Janus metasurface consisting of HWP meta‐atoms can transform *y*‐polarized THz waves into an *x*‐polarized hologram (i.e., a Chinese character of the word “light”). Meanwhile, the QWP meta‐atoms focus *x*‐polarized THz waves into an LCP focal point and an RCP vortex beam. As shown in Figure 6a–h, a *y*‐polarized focal point is observed for the incidence of *y*‐polarized THz waves from the forward direction, which is located after metallic gratings, corresponding to the *y*‐component of the LCP focal point. Additionally, an *x*‐polarized vortex beam is produced, resulting from the *y*‐component of an RCP vortex beam. Therefore, the *y*‐polarized focal point and vortex beam represent the forward manipulation of phase and polarization in the *y*‐polarized channel. Under the backward incidence of *y*‐polarized THz waves, the metallic gratings are transparent, allowing incident THz waves to directly interact with the metasurface. The HWP meta‐atoms transform the incident THz waves into an *x*‐polarized hologram of the Chinese character “light”, as shown in Figure 6i,j. The QWP meta‐atoms convert part of the incident THz waves into an LCP focal point (Figure 6k–n), while the other part transforms into an RCP vortex beam (Figure 6o–r). These calculated results for backward incidence demonstrate the manipulation of phase and polarization in both *x*‐, LCP‐, and RCP‐polarized channels. Generally, our metasurface enables directional phase modulation in the *x*‐, *y*‐, LCP‐, and RCP‐polarized channels. This unique and robust approach allows for designing a plethora of cascaded Janus metasurfaces with directional phase and polarization manipulation in opposite directions, leading to applications in directional imaging as well as direction‐ and polarization‐dependent detection. Compared to traditional bulk THz components, our devices possess the advantages of ultra‐compact footprints and multiple functionalities, suitable for developing miniaturized devices. Although asymmetric phase functions have been investigated in recent years, we extend this to include directional manipulation of polarizations in opposite directions. This simultaneous manipulation of phase and polarization, with different phase and polarization functions, means that more directional functions can be integrated into a metasurface, enhancing capabilities in asymmetric information processing and multiplexing. Additionally, the developed directional devices can asymmetrically encode information by phase and polarization, impacting information security and encryption. Typically, a part of the asymmetric functionalities (i.e., the functions in Figure 3) of our designed metasurfaces can be traditionally realized by putting together a lens, a QWP, and a linear polarizer. However, conventional devices typically should be thick enough and exhibit certain surface topography to ensure the gradual phase accumulation along the propagation direction to realize the desired wave‐manipulating functionalities. The thickness of these traditional THz elements is up to an order of centimeters, resulting in a large and bulk functional device. Since device miniaturization and system integration are two continuing trends for next‐generation integration‐optics applications, our designed directional device opens a new avenue to address the issues. The thickness of the dielectric metasurface and metallic gratings is 0.5 mm and 0.3 µm, while it is 0.5 mm for the spacer (silicon). The total thickness of the device is ≈1 mm, which is much thinner than traditional devices. Therefore, the uniqueness and robustness of our proposed approach may provide an ultra‐compact platform for designing future multifunctional and ultra‐compact devices. In addition, traditional devices cannot be combined to control both phase and polarization directionally.

In conclusion, we have proposed and experimentally demonstrated a novel Janus metasurface platform capable of directionally manipulating phase and polarization. Two types of cascaded Janus metalenses have been designed and fabricated to independently control phase and polarization for directional focusing and polarization conversion. The direction‐ and polarization‐dependent detection and directional imaging were experimentally implemented based on the directional focal points with different polarization states in opposite directions. These cascaded metasurfaces, which enable functionalities in directionally controlling phase and polarization, show potential applications in data storage, information security, and encryption.

## 4 Experimental Section

### Simulation

Full‐wave simulations were conducted using Lumerical. To optimize the unit structure in Figure 2, periodic boundary conditions were applied along the *x* and *y* directions, while a perfect matching layer (PML) was used in the *z*‐direction. For focusing, imaging, and polarization detection, PMLs were employed along the *x*, y, and *z* directions. The gold layer, selected from the material library, had a thickness of 300 nm. The substrate, consisting of silicon with a thickness of 500 µm and a permittivity of *ε* = 3.45, contains dielectric rods. Plane‐wave sources illuminated the designed metasurface, while a 2D monitor detects electric‐field distributions. The computing power is described as follows: Windows 10 operating system (Microsoft) with 2 × Intel(R) Xeon(R) CPU E5‐2696 v4 @2.20 GHz central processing unit (CPU, Intel Inc.), 192 GB of RAM, and a GeForce RTX 4080 Ti graphical processing unit (GPU, Nvidia Inc.).

### Fabrication

A 1000‐µm‐thick intrinsic silicon wafer (<100>, Ω > 5000 ohm) from HF‐Kejing company was cleaned in an ultrasonic bath. Then, an AZP4620 photoresist film was spun onto the silicon wafer with a thickness of 7–8 µm. The photoresist film was baked on a hotplate at 100 °C for ≈1 min. A mask aligner (IMP SF‐100) was used to expose the resist film. After development, the resist film with elliptical pillars was baked at 100 °C for ≈2 min. After that, the silicon wafer was etched by the DRIE (Bosch) process with SF_6_ and C_4_F_8_ for 95 min. The elliptical meta‐atoms were formed after removing the photoresist. For the metallic gratings, the photoresist was spin‐coated on the backside of the silicon wafer. A predesigned mask was used for aligning and exposing the resist film. After metal coating and ultrasonic stripping, the metallic gratings were obtained.

### Experimental Setup

The electric‐field intensity distributions in the experiment were collected using an all‐fiber near‐field scanning THz microscopy system. A femtosecond laser beam with a wavelength of 1560 nm was split into two parts. One laser beam was guided by optical fiber and coupled with a THz emitter to stimulate the THz radiation. The other laser beam was guided by optical fiber to an optical delay line and then coupled into a frequency doubler. A laser beam with a wavelength of 780 nm from the frequency doubler was then coupled with the THz tip/detector for detecting the electric field distributions. The frequency doubler and THz tip were mounted on a 3D translation stage for 3D field detection. The metasurfaces were fixed, while the THz tip was moved to scan the generated electric‐field distributions. For polarization‐dependent imaging, the metasurfaces and THz tip were fixed, while the imaging targets were scanned for imaging.

## Conflict of Interest

The authors declare no conflict of interest.

## Supporting information

Supporting Information

## Data Availability

The data that support the findings of this study are available in the supplementary material of this article.
