# Supplementary material for: Directional Phase and Polarization Manipulation Using Janus Metasurfaces
Source: Adv Sci (Weinh). 2024 Aug 9;11(38):2406571. doi: 10.1002/advs.202406571 (PMC11481181; doi:10.1002/advs.202406571)
Supplement: Supplementary file 1 — Supporting Information [file ADVS-11-2406571-s001.docx]

Supporting Information

Directional Phase and Polarization Manipulation using Janus Metasurfaces

Yiwen Zhou, Teng Zhang, Guannan Wang, Ziqing Guo, Xiaofei Zang^*^, Yiming Zhu*, Fei Ding^*^, Songlin Zhuang

**S1. Principles for Quarter-Wave Plates (QWPs) and Half-Wave Plates (HWPs)**

For an anisotropy meta-atom that is rotated counterclockwise with an angle of $\alpha$, the Jones matrix with the corresponding function can be written as:

(S1)

where$R$is a rotation matrix, and () represents the transmission coefficients of the anisotropy meta-atom under the linearly polarized (LP) incidence. When $t_{xx}=t_{yy}$, $\alpha=\frac{\pi}{4}$, , the electric field of an *x*-polarized beam passing through a metasurface consisting of a variety of meta-atoms with the function of Eq(1) can be expressed as:

(S2)

Similarly, when $t_{xx}=t_{yy}$, $\alpha=\frac{\pi}{4}$, , the electric field of an *x*-polarized beam passing through a metasurface consisting of a variety of meta-atoms with the function of Eq(1) can be expressed as

(S3)

Therefore, when an LP light beam interacts with meta-atoms with the function of Eq. (S2), the circularly polarized (CP) transmitted light can be obtained. When a CP light beam interacts with meta-atoms with the function of Eq. (S3), an orthogonal CP light beam can be obtained.

**S2. Structural Parameters of the Meta-atoms and Metallic Gratings**

**Table S1** The structural parameters of selected QWP meta-atoms

| Label | 1 | 2 | 3 | 4 | 5 | 6 | 7 | 8 |
| --- | --- | --- | --- | --- | --- | --- | --- | --- |
| *L* (μm) | 48 | 51 | 55 | 58 | 42 | 43 | 46 | 48 |
| *W* (μm) | 39 | 40 | 42 | 44 | 22 | 28 | 31 | 34 |

**Table S2** The structural parameters of HWP meta-atoms

| Label | 1 | 2 | 3 | 4 | 5 | 6 | 7 | 8 |
| --- | --- | --- | --- | --- | --- | --- | --- | --- |
| *L* (μm) | 54 | 55 | 55 | 26 | 31 | 34 | 55 | 55 |
| *W* (μm) | 32 | 36 | 37 | 57 | 55 | 59 | 23 | 27 |

**Table S3** The structural parameters of metallic gratings

| 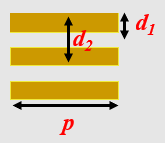 | *d*_1_ | *d*_2_ | *p* |
| --- | --- | --- | --- |
|  | 20 μm | 40 μm | 130 μm |

**S3. Performance of the Single-Focal Janus Metalens under the Illumination of LP THz Waves**

Figure S1 numerically shows the electric-field intensity distributions of the single-focal Janus metalens that can independently and directionally manipulate phase and polarization in opposite directions under the illumination of LP THz waves. For the forward incidence of *x*-polarized THz waves, the calculated electric-field intensity distributions indicate that only one *y*-polarized focal point is observed at *z*= 4.3 mm away from the metallic gratings, as shown in Figure S1a-d. In contrast, when *x*-polarized THz waves are illuminated from the backward direction, no focal point is generated, as demonstrated in Figure S1e-h. For the forward incidence of *y*-polarized THz waves, a *y*-polarized focal point is observed at (0, 0, 4.3 mm) (Figure S1i-l), while an LCP focal point is observed at (0, 0, − 4.3 mm) when *y*-polarized THz waves are illuminated from the backward direction (Figure S1m-p). These two focal points are generated by the same phase function but different polarization states, resulting in directional polarization manipulation.

**
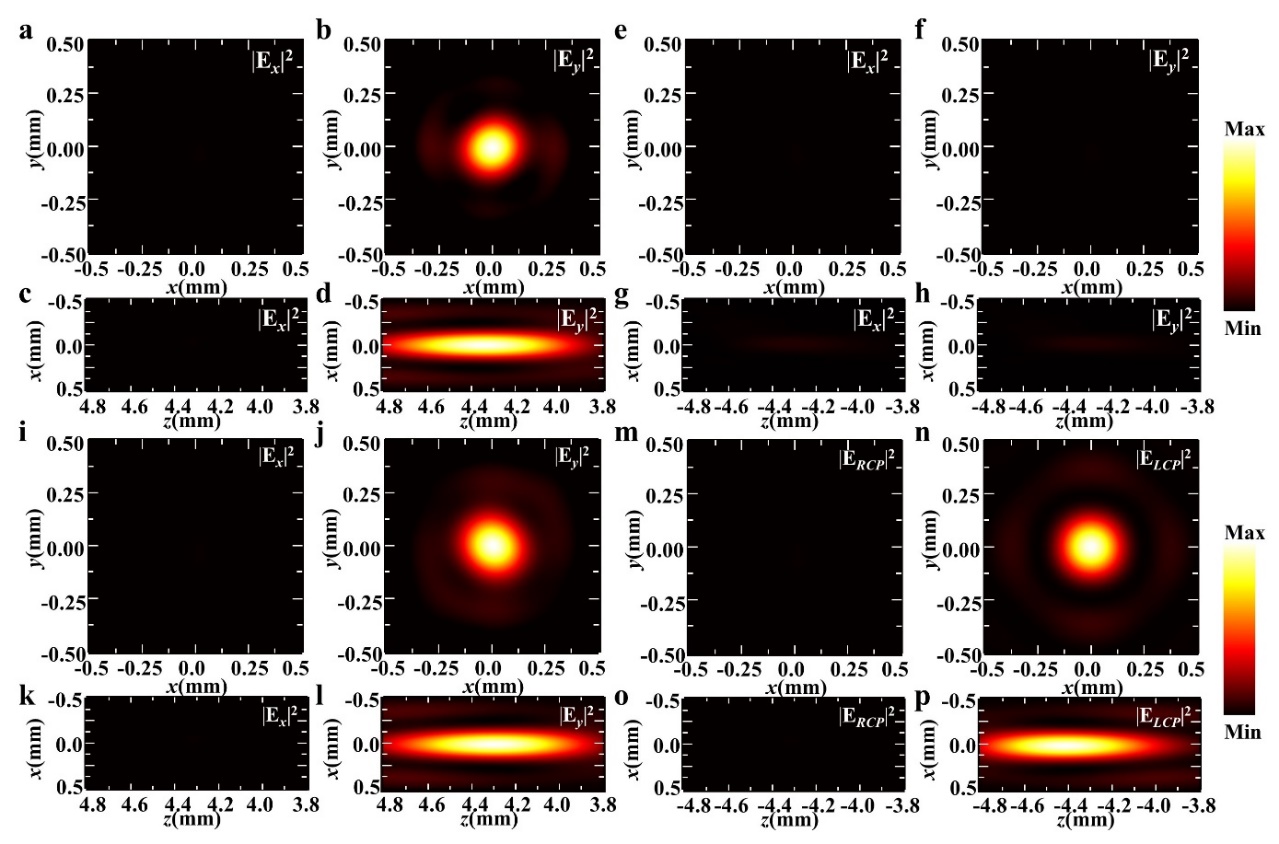
**

**Figure S1.** Single-focal Janus metalens for directional focusing and polarization conversion under the illumination of *x*- and *y*-polarized THz waves. a-d) Simulated electric-field intensity distributions (|*E_x_*|^2^ and | *E_y_*|^2^) at the focal plane (a, b) or the *x*-*z* plane (c, d) for forward incidence of the *x*-polarized THz waves. e-h) Simulated electric-field intensity distributions (|*E_x_*|^2^ and | *E_y_*|^2^) at the focal plane (e, f) or the *x*-*z* plane(g,h) for backward incidence of the *x*-polarized THz waves. i-l) Simulated electric-field intensity distributions (|*E_x_*|^2^ and | *E_y_*|^2^) at the focal plane (i, j) or the *x*-*z* plane (k, l) for forward incidence of the *y*-polarized THz waves. m-p) Simulated electric-field intensity distributions (|*E_x_*|^2^ and | *E_y_*|^2^) at the focal plane (m, n) or the *x*-*z* plane (o, p) for backward incidence of the *y*-polarized THz waves.

**S4. Efficiency of the Single-Focal Janus Metalens**

For the single-focal Janus metasurface, the calculated focusing efficiency is 20.4%, while the measured efficiency is 16.1%, for the incidence of *x*-polarized THz waves from the forward direction. When the incident THz waves are incident from the backward direction, none of a focal point is observed, and thus, the focusing efficiency is zero. For the incidence of *y*-polarized THz waves from the forward direction, a focal point with simulated and measured focusing efficiencies are 20% and 15.8% respectively. When the THz waves are illuminated from the backward direction, the simulated and measured focusing efficiencies are 30.2% and 24.2% respectively. In addition, the measured efficiency is a bit lower than that in simulation, which can be attributed to the fabrication and measurement errors. For the fabrication errors, we can conclude that the fabricated meta-atoms or metallic gratings are mismatched from the predesigned structure sizes. In addition, the large depth-to-width ratio of each meta-atom will inevitably generate the collapse of partial meta-atoms in the fabrication processing. Furthermore, the measured low focusing efficiency can be attributed to the measurement errors including the inhomogeneity and non-perfect signal-to-noise ratio of the THz source.

**Table S4** Focusing efficiency of the single-focal Janus metalens

|  | Simulation | Experiment |
| --- | --- | --- |
| Forward (*x*-polarized incidence) | 20.4% | 16.1% |
| Backward (*x*-polarized incidence) | 0% | 0% |
| Forward (*y*-polarized incidence) | 20% | 15.8% |
| Backward (*y*-polarized incidence) | 30.2% | 24.2% |

**S5. Performance of the Single-Focal Janus Metalens under the Illumination of LCP and RCP THz Waves**

Figure S2 numerically shows the electric-field intensity distributions of the single-focal Janus metalens that can independently and directionally manipulate phase and polarization in opposite directions. As shown in Figure S2a-d for the forward incidence of LCP THz waves, no focal point is generated. In contrast, when LCP THz waves are illuminated from the backward direction, an LCP focal point is observed at (0, 0, − 4.3 mm), as demonstrated in Figure S2e-h. For the incidence of LCP THz waves in opposite directions, only a one-way focal point is generated, demonstrating the directional manipulation of phase. When RCP THz waves are illuminated from the forward direction, a *y*-polarized focal point is observed at (0, 0, 4.3 mm) (Figure S2i-l), while an LCP focal point is observed at (0, 0, − 4.3 mm) under the illumination of RCP THz waves from the backward direction (Figure S2m-p). These two focal points are generated by the same phase function but different polarization states, resulting in the directional polarization manipulation.


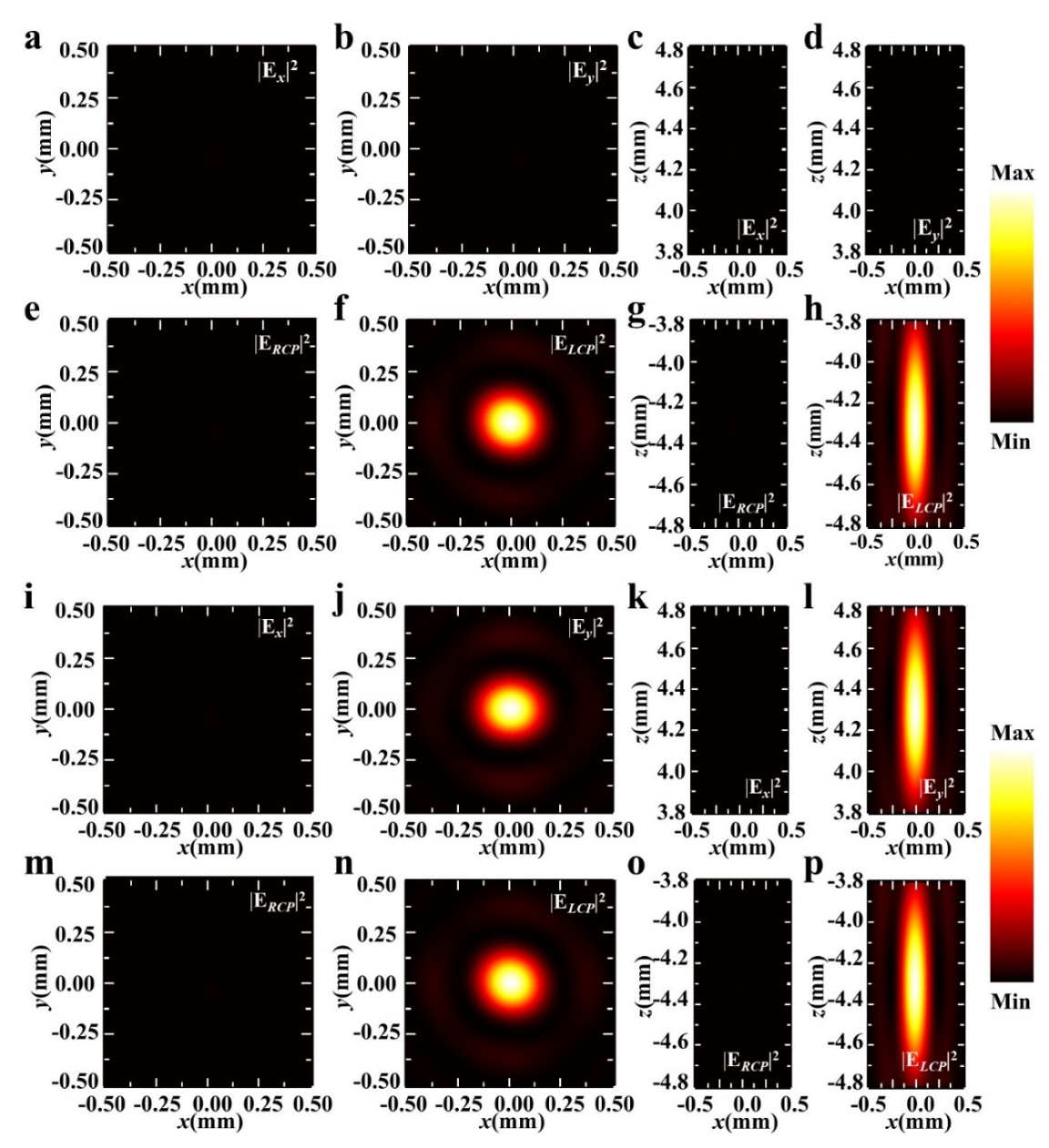


**Figure S2.** Single-focal Janus metalens for directional focusing and polarization conversion under the illumination of LCP and RCP THz waves. a-d) Simulated electric-field intensity distributions (|*E_x_*|^2^ and | *E_y_*|^2^) at the focal plane (a, b) or the *x*-*z* plane (c, d) for forward incidence of the LCP THz waves. e-h) Simulated electric-field intensity distributions (|*E_LCP_*|^2^ and | *E_RCP_*|^2^) at the focal plane (e, f) or the *x*-*z* plane (g, h) for backward incidence of the LCP THz waves. i-l) Simulated electric-field intensity distributions (|*E_x_*|^2^ and | *E_y_*|^2^) at the focal plane (i, j) or the *x*-*z* plane (k, l) for forward incidence of the RCP THz waves. m-p) Simulated electric-field intensity distributions (|*E_LCP_*|^2^ and | *E_RCP_*|^2^) at the focal plane (m, n) or the *x*-*z* plane (o, p) for backward incidence of the RCP THz waves.

**S6. Performance of a Janus Metalens Consisting of HWPs and Metallic Gratings**

To validate the versatility of our concept, we designed a single-focal Janus metalens consisting of HWPs and metallic gratings for directional focusing and polarization conversion. For the forward incidence of *x*-polarized THz waves, a *y*-polarized focal point is observed at *z* = 4.3 mm away from the metallic gratings (Figure S3a,c-f), while no focal point is generated for *x*-polarized THz waves incident from the backward direction (Figure S3b,g-j). For the forward case, the *x*-polarized THz waves interact with the HWP meta-atoms and are transformed into a *y*-polarized focusing beam. Thus, the *y*-polarized focusing beam can transmit through the metallic gratings, leading to a *y*-polarized focal point. When the *x*-polarized THz waves illuminate from the backward direction, they are completely reflected by the metallic gratings without forming a focal point. When the *y*-polarized THz waves illuminate from the forward direction, an *x*-polarized focusing beam is first formed and then reflected by the metallic gratings. Therefore, no focal point is formed after the metallic gratings, as shown in Figure S3k,m-p. For the incidence of *y*-polarized THz waves from the backward direction, a focal point is generated at *z* = − 4.3 mm, as shown in Figure S3l,q-t.


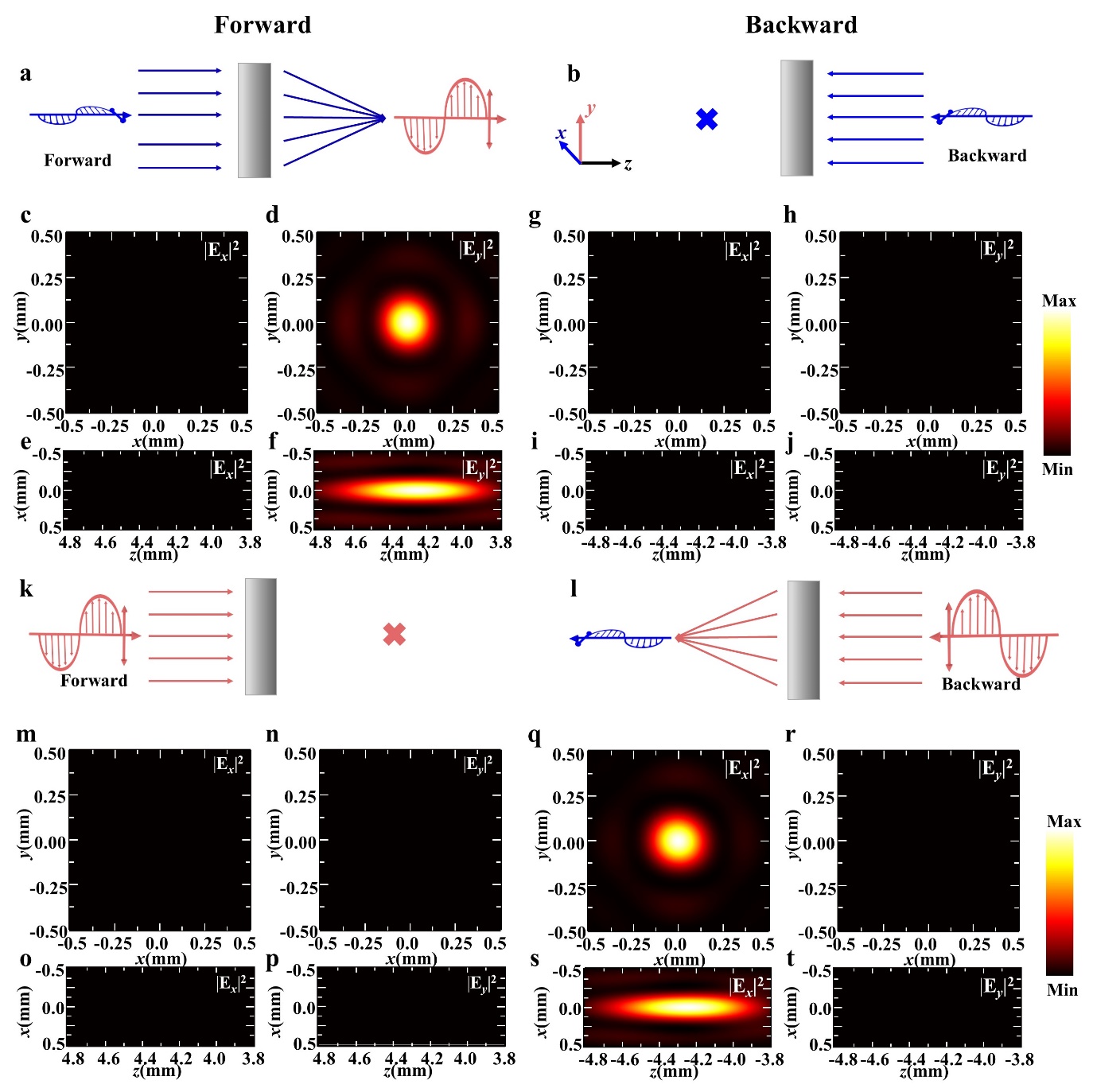


**Figure S3.** Single-focal Janus metalens consisting of HWPs and metallic gratings for directional focusing. a, b) Schematics of one-way focusing under the incidence of *x*-polarized THz waves. c-f) The calculated electric-field intensity distributions (|*E_x_*|^2^ and | *E_y_*|^2^) at the focal plane (c, d) or the *x*-*z* plane (e, f) for forward incidence of *x*-polarized THz waves. g-j) The calculated electric-field intensity distributions (|*E_x_*|^2^ and | *E_y_*|^2^) at the focal plane (g, h) or the *x*-*z* plane (i, j) for backward incidence of *x*-polarized THz waves. k, l) Schematics of focusing and directional polarization manipulation for the forward (k) and backward (l) incidence of *y*-polarized THz waves. m-p) The calculated electric-field intensity distributions (|*E_x_*|^2^ and | *E_y_*|^2^) at the focal plane (m,n) or the *x*-*z* plane (o, p) for forward incidence of *y*-polarized THz waves. q-t) The calculated electric-field intensity distributions (|*E_x_*|^2^ and | *E_y_*|^2^) at the focal plane (q, r) or the *x*-*z* plane (s, t) for backward incidence of *y*-polarized THz waves.

**S7. Efficiency of the Dual-Focal Janus Metalens**

For the forward incidence, the simulated and measured focusing efficiencies are 13.1% and 10.4%, respectively. The measured efficiency is a bit lower than that in simulation, which can be attributed to the fabrication and measurement errors. When the incident THz wave is switched into the backward direction, two focal points (*i.e.*, an LCP focal point and an *x*-polarized focal point) are observed after the meta-atoms. The calculated focusing efficiency is 26% and the measured focusing efficiency is 20.8%. The measured efficiency is a little lower than that of the simulated result. It should be noted that the forward focal point is only contributed to the HWP meta-atoms while the backward two focal points are generated from both HWP and QWP meta-atoms, and thus, the focusing efficiency in the forward direction is lower than that in the backward direction.

**Table S5** Focusing efficiency of the dual-focal Janus metalens

|  | Simulation | | | Experiment |
| --- | --- | --- | --- | --- |
| Forward (*y*-polarized incidence) | | | 13.1% | 10.4% |
| Backward (*y*-polarized incidence) | | 26% | | 20.8% |

**S8. Performance of the Dual-Focal Janus Metalens under the Illumination of *y*-Polarized THz Waves**

The calculated characteristics of the dual-focal Janus metalens under the illumination of *y*-polarized THz waves are shown in Figure S4. For the forward incidence of *y*-polarized THz waves, only one *y*-polarized focal point at (−1.5 mm, 0, 4.3 mm) is observed, as demonstrated in Figure S4a-d. In contrast, when *y*-polarized THz waves are illuminated from the backward direction, two focal points are generated and at (−1.5 mm, 0, −4.3 mm) and (1.5 mm, 0, 4.3 mm), respectively. For the backward incidence of *y*-polarized THz waves, the metallic gratings are transparent, allowing the transmitted THz waves to be converted into an LCP focal point (Figure S4e-h) and an *x*-polarized focal point (Figure S4i-l) due to the QWP and HWP meta-atoms, respectively.

**
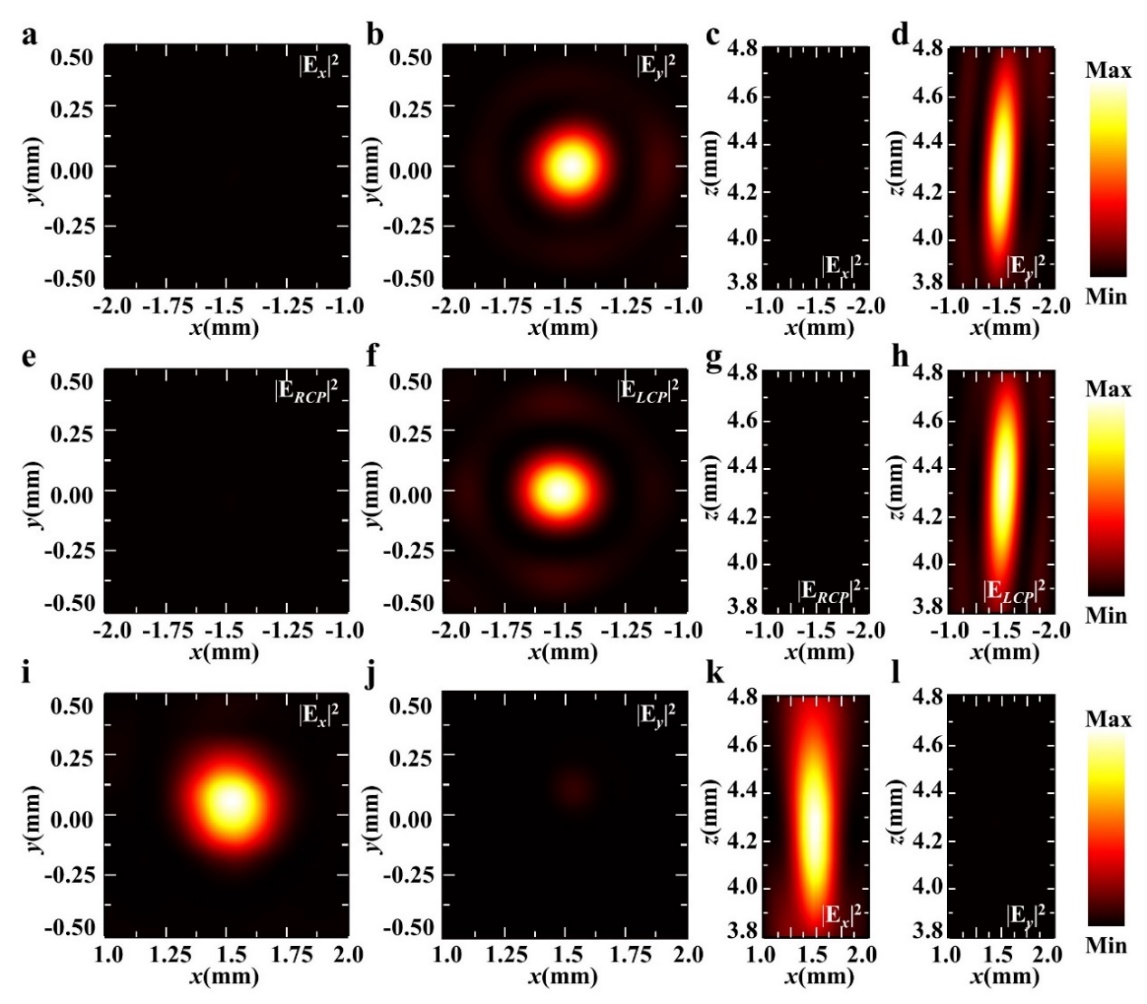
**

**Figure S4.** Dual-focal Janus metalens for directional focusing and polarization conversion under the illumination of *y*-polarized THz waves. a-d) Simulated electric-field intensity distributions (|*E_x_*|^2^ and | *E_y_*|^2^) at the focal plane (a, b) or the *x*-*z* plane (c, d) for forward incidence of the *y*-polarized THz waves. e-h) Simulated electric-field intensity distributions (|*E_LCP_*|^2^ and | *E_RCP_*|^2^) at the focal plane (e, f) or the *x*-*z* plane (g, h) for backward incidence of the *y*-polarized THz waves. i-l) Simulated electric-field intensity distributions (|*E_LCP_*|^2^ and | *E_RCP_*|^2^) at the focal plane (i, j) or the *x*-*z* plane (k, l) for backward incidence of the *y*-polarized THz waves.

**S9. Performance of the Dual-Focal Janus Metalens under the Illumination of *x*-Polarized, LCP, and RCP THz Waves**

Figure S5 numerically demonstrates the electric-field intensity distributions of the dual-focal Janus metalens that can simultaneously and directionally manipulate phase and polarization in opposite directions. As shown in Figure S5a,c, two *y*-polarized focal points are generated at (−0.5 mm, 0, 4.3 mm) and (0.5 mm,0, 4.3 mm), respectively, for the incidence of *x*-polarized THz waves from the forward direction. In contrast, when *x*-polarized THz waves are illuminated from the backward direction, no focal point is generated, as demonstrated in Figure S5b,d. For the forward incidence of *x*-polarized THz waves, only two one-way focal points are generated, demonstrating the directional phase manipulation. When LCP THz waves are illuminated from the forward direction, a *y*-polarized focal point is observed at (0.5 mm, 0, 4.3 mm) (Figure S5e,g). In contrast, under the backward illumination of LCP THz waves, an LCP focal point and an *x*-polarized focal point are observed at (−0.5 mm, 0, −4.3 mm) and (0.5 mm, 0, −4.3 mm), respectively (Figure S5f,h). The focal point at (−0.5 mm, 0, −4.3 mm) is generated by the phase function from the HWP meta-atoms, while the focal point at (0.5 mm, 0, −4.3 mm) is contributed by the QWP meta-atoms. When RCP THz waves are illuminated from the forward direction, two focal points polarized along the *y*-axis are observed at (0.5 mm, 0, 4.3 mm) and (0.5 mm, 0, −4.3 mm) (Figure S5i,k). Under the backward illumination of RCP THz waves, an LCP focal point and an *x*-polarized focal point are observed at (−0.5 mm, 0, −4.3 mm) and (0.5 mm, 0, −4.3 mm) (Figure S5j,l). In comparison with Figure S5e,k and Figure S5f,l, the simultaneous manipulation of phase and polarization (*i.e.*, directional phase and polarization functions) can be realized by the illumination of LCP/RCP THz waves from opposite directions.


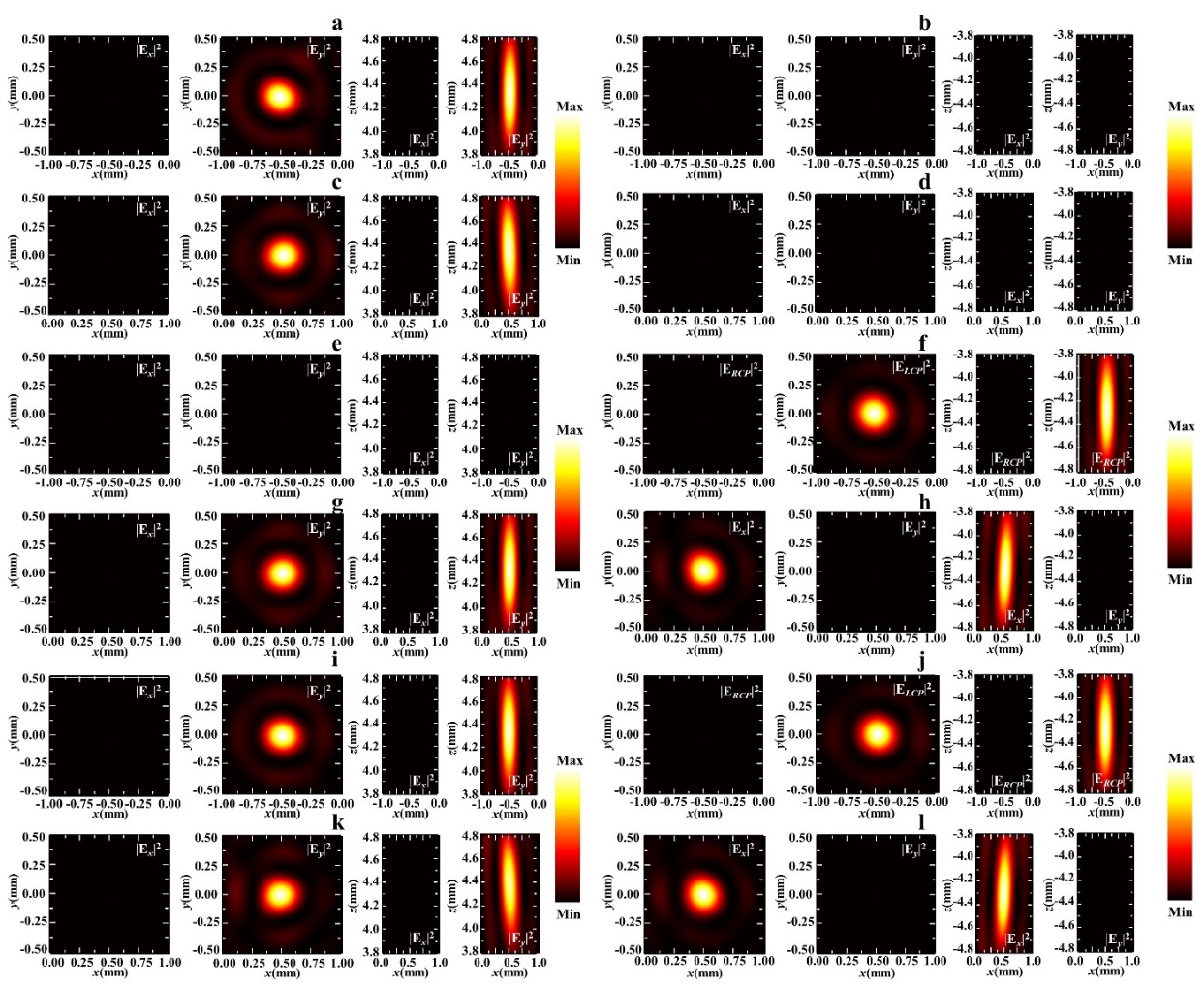


**Figure S5.** Dual-focal Janus metalens for directional focusing and polarization conversion under the illumination of *x*-polarized, LCP and RCP THz waves. a, c) Simulated electric-field intensity distributions (|*E_x_*|^2^ and |*E_y_*|^2^) at the focal plane or the *x*-*z* plane for forward incidence of the *x-*polarized THz waves. e, g) Simulated electric-field intensity distributions (|*E_x_*|^2^ and |*E_y_*|^2^) at the focal plane or the *x*-*z* plane for forward incidence of the LCP THz waves. i, k) Simulated electric-field intensity distributions (|*E_x_*|^2^ and |*E_y_*|^2^) at the focal plane or the *x*-*z* plane for forward incidence of the RCP THz waves. b, d) Simulated electric-field intensity distributions (|*E_x_*|^2^ and |*E_y_*|^2^) at the focal plane or the *x*-*z* plane for backward incidence of the *x-*polarized THz waves. f, h) Simulated electric-field intensity distributions (|*E_x_*|^2^, |*E_y_*|^2^, |*E_LCP_*|^2^ and |*E_RCP_*|^2^) at the focal plane or the *x*-*z* plane for backward incidence of the LCP THz waves. j, l) Simulated electric-field intensity distributions (|*E_x_*|^2^ and | *E_y_*|^2^, |*E_LCP_*|^2^ and |*E_RCP_*|^2^) at the focal plane or the *x*-*z* plane for backward incidence of the RCP THz waves.

**S10. Janus Metasurface Consisting of QWP Meta-Atoms for Directionally Controlling a Vortex Beam**

Figure S6 numerically illustrates the electric-field intensity distributions of a Janus metasurface for directionally controlling vortex beams. As shown in Figure S6a for the forward incidence of *x*-polarized THz waves, a *y*-polarized vortex beam with a topological charge of *l* = 1 is observed at (0, 0, −4.3 mm). In contrast, when *x*-polarized THz waves are illuminated from the backward direction, no vortex beam is generated, as demonstrated in Figure S6b. For the incidence of *x*-polarized THz waves in the forward and backward directions, only a one-way vortex beam is generated, demonstrating the directional phase manipulation. When *y*-polarized THz waves are illuminated from the forward direction, a *y*-polarized vortex beam is observed at (0, 0, 4.3 mm) (Figure S6c), while an LCP vortex beam is observed at (0, 0, −4.3 mm) under the illumination of *y*-polarized THz waves from the backward direction (Figure S6d), resulting in the directional polarization manipulation. As shown in Figure S6e, no vortex beam is generated for the forward incidence of LCP THz waves. In contrast, an LCP vortex beam is observed at (0, 0, −4.3 mm) when LCP THz waves are illuminated from the backward direction, as shown in Figure S6f. For the incidence of LCP THz waves in two opposite directions, only a one-way vortex beam is generated. When RCP THz waves are illuminated from the forward direction, a *y*-polarized vortex beam is observed at (0, 0, 4.3 mm) (Figure S6g), while an LCP vortex beam is observed at (0, 0, −4.3 mm) under the illumination of RCP THz waves from the backward direction (Figure S6h). These two vortex beams are generated by the same phase function but different polarization states, demonstrating the directional polarization manipulation.


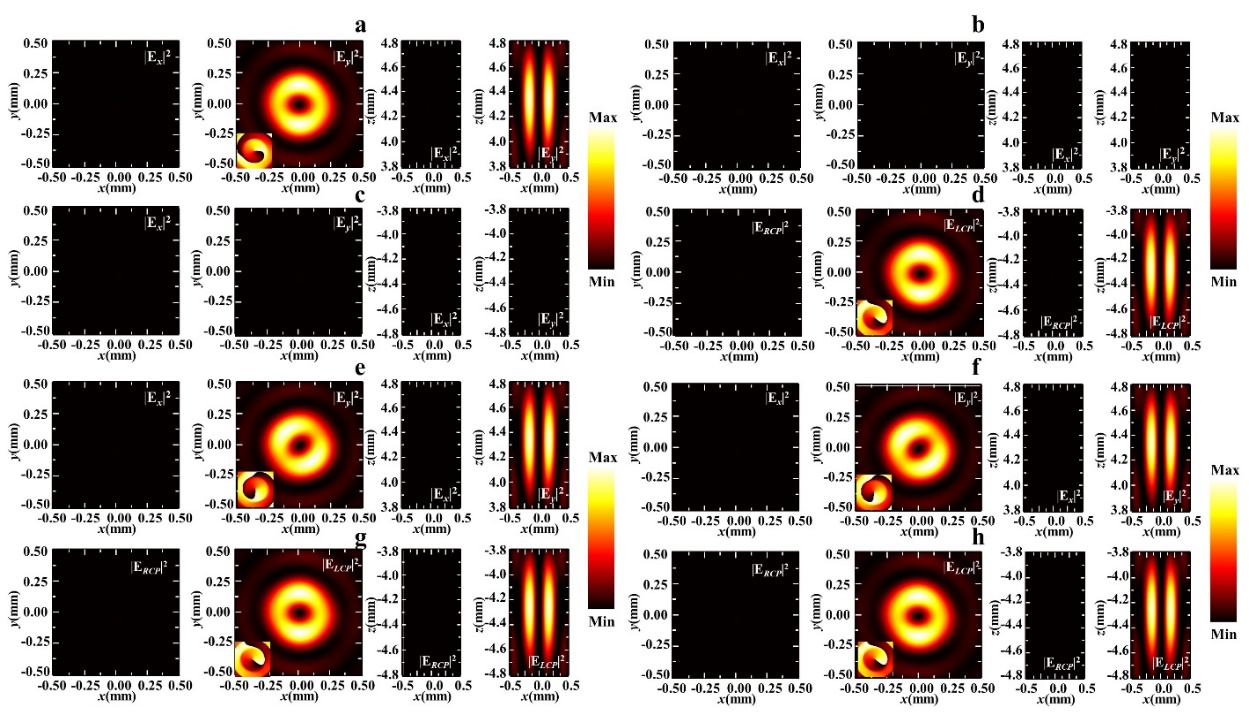


**Figure S6.** Janus metasurface for directionally controlling a vortex beam. a) Simulated electric-field intensity and phase distributions (|*E_x_*|^2^ and |*E_y_*|^2^) at *z* = 4.3 mm or the *x*-*z* plane for forward incidence of the *x-*polarized THz waves. b) Simulated electric-field intensity distributions (|*E_x_*|^2^ and |*E_y_*|^2^) at *z* = 4.3 mm or the *x*-*z* plane for backward incidence of the *x-*polarized THz waves. c) Simulated electric-field intensity and phase distributions (|*E_x_*|^2^ and |*E_y_*|^2^) at *z* = 4.3 mm or the *x*-*z* plane for forward incidence of the *y-*polarized THz waves. d) Simulated electric-field intensity distributions (|*E_LCP_*|^2^ and |*E_RCP_*|^2^) at *z* = 4.3 mm or the *x*-*z* plane for backward incidence of the *y-* polarized THz waves. e) Simulated electric-field intensity and phase distributions (|*E_x_*|^2^ and |*E_y_*|^2^) at *z* = 4.3 mm or the *x*-*z* plane for forward incidence of the LCP THz waves. f) Simulated electric-field intensity distributions (|*E_LCP_*|^2^ and |*E_RCP_*|^2^) at *z* = 4.3mm or the *x*-*z* plane for backward incidence of the LCP THz waves. g) Simulated electric-field intensity and phase distributions (|*E_x_*|^2^ and |*E_y_*|^2^) at *z* = 4.3 mm or the *x*-*z* plane for forward incidence of the RCP THz waves. h) Simulated electric-field intensity distributions (|*E_LCP_*|^2^ and |*E_RCP_*|^2^) at *z* = 4.3 mm or the *x*-*z* plane for backward incidence of the RCP THz waves.

**S11. Janus Metasurface Consisting of QWP and HWP Meta-Atoms for Directionally Controlling Vortex Beams**

Figure S7 presents the numerical simulations of a Janus metasurface that can simultaneously and directionally control the phase and polarization of vortex beams in opposite directions. As shown in Figure S7a,c for the forward incidence of *x*-polarized THz waves, two *y*-polarized vortex beams are generated at (−0.5 mm, 0, 4.3 mm) and (−0.5 mm, 0, 4.3 mm) respectively. In contrast, when *x*-polarized THz waves are illuminated from the backward direction, no vortex beam is generated, as demonstrated in Figure S7b,d, demonstrating the directional manipulation of phase and the generation of one-way vortex beams. As shown in Figure S7e,g for the forward incidence of *y*-polarized THz waves, a *y*-polarized vortex beam is generated at (−0.5 mm, 0, 4.3 mm). In contrast, when the *y*-polarized THz waves are illuminated from the backward direction, an LCP vortex beam and a *x*-polarized vortex beam are observed at (−0.5 mm, 0, −4.3 mm) and (0.5 mm, 0, −4.3 mm) respectively, as shown in Figure S7f.h. The numerical simulations in Figure S7f,h demonstrate that our designed metasurface enables simultaneous and directional control over the phase and polarization. When LCP THz waves are illuminated from the forward direction, a *y*-polarized vortex beam is observed at (0.5 mm, 0, 4.3 mm) (Figure S7i,k), while an LCP vortex beam and an *x*-polarized vortex beam are observed at (−0.5 mm, 0, −4.3 mm) and (0.5 mm, 0, −4.3 mm), respectively, under the illumination of LCP THz waves from the backward direction (Figure S7j,l). When RCP THz waves are illuminated from the forward direction, two *y*-polarized vortex beams are observed at (0.5 mm, 0, 4.3 mm) and (0.5 mm, 0, −4.3 mm) (Figure S7m,o), while an LCP vortex beam and a *x*-polarized vortex beam are observed at (−0.5 mm, 0, −4.3 mm) and (0.5 mm, 0, −4.3 mm), respectively, under the illumination of RCP THz waves from the backward direction (Figure S7n,p).


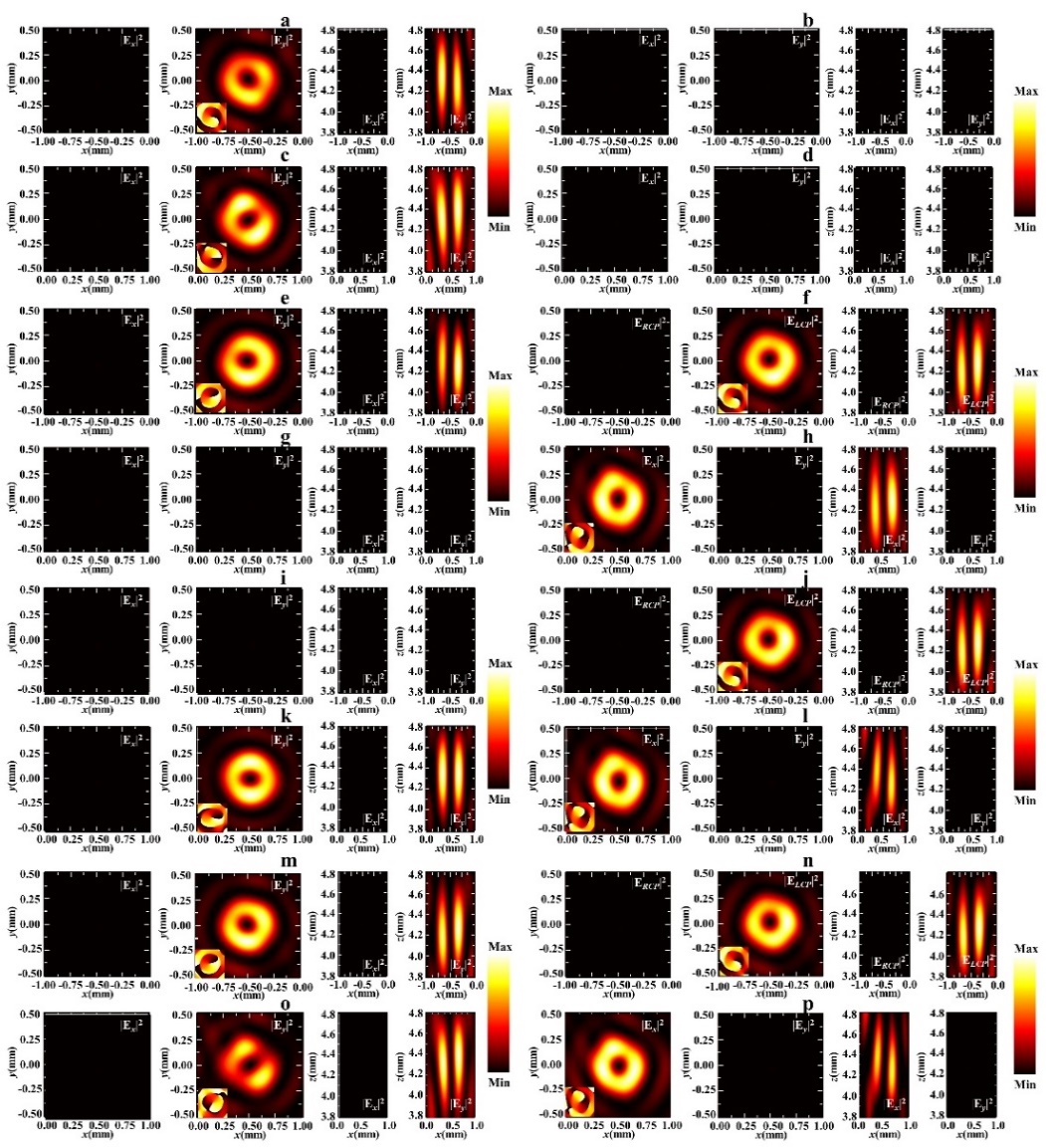


**Figure S7.** Janus metasurface for directionally controlling vortex beams. **a, c)** Simulated electric-field and phase intensity distributions (|*E_x_*|^2^ and |*E_y_*|^2^) at *z* = 4.3 mm or the *x*-*z* plane for forward incidence of the *x-*polarized THz waves. **e, g)** Simulated electric-field intensity and phase distributions (|*E_x_*|^2^ and |*E_y_*|^2^) at *z* = 4.3 mm or the *x*-*z* plane for forward incidence of the *y-*polarized THz waves. (i, k) Simulated electric-field intensity and phase distributions (|*E_x_*|^2^ and |*E_y_*|^2^) at *z* = 4.3 mm or the *x*-*z* plane for forward incidence of the LCP THz waves. **m, o)** Simulated electric-field intensity and phase distributions (|*E_x_*|^2^ and |*E_y_*|^2^) at *z* = 4.3 mm or the *x*-*z* plane for forward incidence of the RCP THz waves. **b, d)** Simulated electric-field intensity distributions (|*E_x_*|^2^ and |*E_y_*|^2^) at *z* = 4.3 mm or the *x*-*z* plane for backward incidence of the *x-*polarized THz waves. **f, h)** Simulated electric-field intensity and phase distributions (|*E_x_*|^2^, |*E_y_*|^2^, |*E_LCP_*|^2^ and |*E_RCP_*|^2^) at *z* = 4.3 mm or the *x*-*z* plane for backward incidence of the *y-*polarized THz waves**. j, l)** Simulated electric-field intensity and phase distributions (|*E_x_*|^2^ and |*E_y_*|^2^, |*E_LCP_*|^2^ and |*E_RCP_*|^2^) at *z* = 4.3 mm or the *x*-*z* plane for backward incidence of the LCP THz waves. (n, p) Simulated electric-field intensity and phase distributions (|*E_x_*|^2^ and |*E_y_*|^2^, |*E_LCP_*|^2^ and |*E_RCP_*|^2^) at *z* = 4.3 mm or the *x*-*z* plane for backward incidence of the LCP THz waves.

**S12. Efficiency of the imaging**

For the single imaging of “T”, the simulated efficiency is 10.34%, while the measured efficiency is 6.2%. For the dual imaging of “T T”, the simulated efficiency is 16.66%, while the measured efficiency is 10.37%. The measured efficiency of imaging is lower than that of the simulated results, which can be attributed to fabrication and measurement errors.

**Table S6** Efficiency of the dual-focal Janus metalens for imaging

|  | Simulation | | Experiment |
| --- | --- | --- | --- |
| Forward (a single image “T”) | | 10.34% | 6.2% |
| Backward (dual images “T T”) | 16.66% | | 10.37% |
